# Different Molecular/Behavioral Endophenotypes in C57BL/6J Mice Predict the Impact of OX_1_ Receptor Blockade on Binge-Like Ethanol Intake

**DOI:** 10.3389/fnbeh.2017.00186

**Published:** 2017-10-10

**Authors:** Manuel Alcaraz-Iborra, Francisco Navarrete, Elisa Rodríguez-Ortega, Leticia de la Fuente, Jorge Manzanares, Inmaculada Cubero

**Affiliations:** ^1^Laboratorio de Psicobiología, Departamento de Psicologia, Universidad de Almería, Almería, Spain; ^2^Instituto de Neurociencias, Consejo Superior de Investigaciones Científicas (CSIC), Universidad Miguel Hernández de Elche, Elche, Spain; ^3^Institute of Biomedical Sciences, Universidad Autonoma de Chile, Santiago de Chile, Chile

**Keywords:** anxiety, neophobia, impulsivity/compulsivity, intermittent ethanol drinking in the dark, orexin, endophenotype

## Abstract

Ethanol (EtOH) research has focused on stages of dependence. It is of paramount importance to more deeply understand the neurobehavioral factors promoting increased risk for EtOH binge drinking during the early stages of the addiction cycle. The first objective of this study was to evaluate whether C57BL/6J mice showing high drinking in the dark (DID) exhibit neurobehavioral traits known to contribute to EtOH binge-drinking disorders. Comparing high vs. low drinkers (HD/LD), we evaluated different types of basal anxiety-like responses, EtOH preference and sensitivity to the reinforcing properties of EtOH, and basal mRNA expression of the OX1/OX2 receptors (OX1r/OX2r) within the prefrontal cortex (PFC) and the nucleus accumbens (NAcc). Additionally, we tested binge drinking by LD/HD in response to a selective OX1r antagonist following intermittent episodes of DID (iDID). We report that DID consistently segregates two neurobehavioral endophenotypes, HD vs. LD, showing differences in neophobia and/or impulsivity/compulsivity traits. Additionally, HD mice show decreased basal OX1r and OX2r mRNA expression within the NAcc and elevated OX1r within the PFC. Exposure to several intermittent episodes of EtOH DID triggered a rapid increase in EtOH intake over time in LD mice matching that observed in HD mice. Despite HD/LD endophenotypes did not show differences in EtOH intake, they still predicted the response to a pharmacological challenge with a selective OX1r antagonist. The present data underscore the relevance of HD/LD endophenotypes stemming from DID procedures for exploring neurobehavioral processes underlying the early stages of the addiction cycle and EtOH binge-drinking disorders.

## Introduction

Approximately 4.9% of the world’s adult population is believed to suffer from Alcohol Use Disorders (AUDs; Gowing et al., 2015), which rank among the leading public health problems worldwide. Drug and ethanol (EtOH) addiction has been conceptualized as a chronic disorder that involves elements of impulsivity and compulsivity, yielding a composite addiction cycle comprising three progressive stages ultimately leading to the pathological addiction state (Koob, 2013): (1) a binge-intoxication phase driven by the rewarding properties of the drug; (2) a withdrawal phase; and (3) a preoccupation-anticipation phase that precedes renewed drug intake, governed by negative reinforcement and increased sensitivity in the brain stress system (Koob, 2013).

Traditionally, drug and EtOH research has focused on later, dependent stages by employing models of EtOH dependence (for a review, see Yardley and Ray, 2017) and EtOH relapse (for a review, see Vengeliene et al., 2014). However, the early stages of the addiction cycle governed by binge drinking prior to the onset of dependence have received far less attention. Frequent and intermittent EtOH binge drinking is a typical pattern of excessive EtOH consumption for experiencing intoxication, exhibited during early stages of the addiction cycle (Crabbe et al., 2011), that represents a substantial risk factor predicting the development of AUDs in vulnerable organisms (Courtney and Polich, 2009; Crabbe et al., 2011; Thiele and Navarro, 2014). Thus, it is of paramount importance to understand the neurochemical and behavioral mechanisms that control binge drinking in non-dependent organisms, as such knowledge may provide insight into novel pharmacological and behavioral therapeutic approaches that may protect vulnerable individuals from progressing to the point of EtOH dependence (Thiele and Navarro, 2014). In this context, a mouse model of binge-like EtOH drinking, “drinking in the dark” (DID; Rhodes et al., 2005), has been useful for studying the neurobiology underlying the early stages in the cycle of addiction and the transition to EtOH dependence (Cox et al., 2013; for a review, see Thiele and Navarro, 2014).

In EtOH research, there is consistent evidence suggesting that some premorbid behavioral traits and psychobiological risk factors, such as high anxiety and stress, increased reactivity to the rewarding properties of EtOH or enhanced impulsivity/compulsivity, might significantly increase vulnerability to binge EtOH intake, favoring subsequent AUDs (Wand, 2005; Iacono et al., 2008; Casey and Jones, 2010). On the other hand, the comparative analysis of high vs. low EtOH drinkers (HD/LD), as subpopulations spontaneously emerging when exposed to voluntary EtOH drinking, has represented one successful experimental approach in this field for addressing the existence of neurobehavioral factors favoring vulnerability to excessive EtOH consumption (Mardones and Segovia-Riquelme, 1983; Barson et al., 2013). Given the relevance of a deeper understanding of neurobehavioral factors promoting increased risk for binge-drinking during the early stages of the addiction cycle, the first objective in the study was to evaluate whether HD mice might exhibit behavioral traits known to contribute to AUDs (Wand, 2005; Iacono et al., 2008; Casey and Jones, 2010). Thus, we comparatively evaluated in HD vs. LD for basal anxiety-like responses. It is worthy to note that different types of anxiety-related behavior have been distinguished in rodents (Griebel et al., 1993; Kopp et al., 1999; van Gaalen and Steckler, 2000) and different behavioral principles seem to underlie the design of the various anxiety tasks. Thus, different tasks show different sensitivities for the anxiolytic- or anxiogenic-like actions of drugs, and task-dependent effects have been demonstrated (van Gaalen and Steckler, 2000). Therefore, testing HD/LD endophenotypes using a set of different anxiety paradigms would be advantageous. Under this framework, different types of basal anxiety-like responses were included as measured by the elevated plus maze (EPM), the Novel Object Exploration and Marble Burying tests. Finally, given that differences in sensitivity to the EtOH reinforcing and toxic properties regulate EtOH consumption, we tested HD/LD mice for conditioned place preference (CPP) and EtOH preferences under the 2-bottle free choice task.

During recent years, a growing experimental pharmacological and genetic literature has consistently supported the involvement of cerebral peptides in excessive EtOH consumption, thus providing new potential molecular targets for improving current therapeutic approaches to AUDs (Ubaldi et al., 2013; Khoo and Brown, 2014). In this regard, a role for Orexin (OX) in EtOH seeking (Lawrence et al., 2006; Brown and Lawrence, 2013; Martin-Fardon and Weiss, 2014), EtOH self-administration (Dhaher et al., 2010; Jupp et al., 2011; Brown et al., 2013) and binge-like EtOH drinking (Carvajal et al., 2015; Olney et al., 2015) has recently been demonstrated. In a 2-bottle free choice paradigm, peripheral administration of the selective OX1r antagonist SB-334867 significantly reduced voluntary EtOH consumption in Sprague-Dawley rats showing high, but not low, EtOH preference (Moorman and Aston-Jones, 2009). Further, OX1r antagonism/blockade reduced voluntary EtOH self-administration in rats selectively bred for high EtOH preference (Anderson et al., 2014) and EtOH binge-drinking in C57BL/6J mice (Anderson et al., 2014; Carvajal et al., 2015; Olney et al., 2015). In this framework, our second goal was to compare HD and LD subpopulations stemming from a DID procedure in terms of their basal mRNA expression of OX1/OX2 receptors (OX1r/OX2r) within two brain regions involved in EtOH addiction and receiving OX projections: the prefrontal cortex (PFC) and nucleus accumbens (NAcc). Given that real time PCR is a very sensitive technique capable to detect small mRNA level changes whereas protein expression evaluation methods (Western Blot, Immunohistochemistry, etc.) are less sensitive and often are not able to detect small protein expression changes, we have focused on gene expression analyses.

It has been proposed that binge-like EtOH drinking with DID procedures may be modulated by transient changes in central neurochemical systems that arise from high BECs in non-dependent animals. Such initially transient neuroplastic changes might become consolidated through repeated episodes of binge-like drinking, contributing to the transition to addictive states (Cox et al., 2013; Thiele and Navarro, 2014). Consistent with this idea, we have recently proposed that repetitive episodes of binge-like consumption of a rewarding stimulus in non-dependent organisms may enhance OX transmission in vulnerable organisms in a positive loop that favors the transition to addiction, from non-dependent/impulse-driven binge consumption to compulsion-driven consumption (Alcaraz-Iborra and Cubero, 2015). Under this hypothesis, excessive OX activity might work as a neurobiological mechanism predisposing the transition from EtOH binge drinking to EtOH addiction. Thus, we predict a progressive increase in OX activity in vulnerable mice exposed to continued EtOH consumption (Alcaraz-Iborra and Cubero, 2015). The third aim in the study tested potential changes in the OX system following intermittent episodes of DID (iDID). To that end, we compared EtOH binge consumption in HD/LD mice exposed to prolonged iDID and their responses to the selective OX1r antagonist SB-334867.

## Materials and Methods

### Animals and Housing

Four independent cohorts of 8-week-old adult male C57BL/6J mice (Charles River Laboratories, S.A., Spain) were tested in series of behavioral, pharmacological and molecular tasks (*n* = 92). Cohort 1 (*n* = 24): one single 2 h episode of binge-like EtOH drinking followed by 10 days with no manipulation and then brain extraction for mRNA analysis; Cohort 2 (*n* = 12): one single 2 h episode of binge-like EtOH drinking followed by 10 days with no manipulation and then EPM; Cohort 3 (*n* = 32): one single 2 h episode of binge-like EtOH drinking followed by 10 days with no manipulation and then CPP (*n* = 16), 2-bottle free choice task (*n* = 16), marble burying and novel object exploration (*n* = 32); Cohort 4 (*n* = 24): intermittent DID aimed at evaluating the effect of anOX1r antagonist, SB-334867. Mice weighed 20–25 g on arrival and were housed individually in polycarbonate cages with stainless steel wire mesh lids and sawdust covering the floor. Animals were allowed to acclimate to the housing environment for 1 week before any experimental procedures. The animal house was kept at approximately 21 ± 2°C in a 12:12 h dark/light schedule (lights off 8 am–8 pm). Animals had *ad libitum* access to chow and water, and all experimental procedures were conducted during the animal’s activity phase (dark period). Behavioral procedures were approved by the Bioethical Animal Care Committee at the University of Almeria, Spain, and they were consistent with the animal care guidelines established by the Spanish Royal Decree 53/2013 for reducing animal pain and discomfort.

#### Drugs

The OX1r antagonist SB-334867 (SB: 1-(2-methylbenzoxazol-6-yl)-3-[1,5]naphthyridin-4-yl urea hydrochloride, Tocris, Bristol, UK) was suspended in 1.5% dimethyl sulfoxide (DMSO), 20% 2-hydroxypropyl-beta-cyclodextrin (HBC) and sterile water. For the pharmacological challenge, 5 mg/kg of SB was given in a volume of 10 ml/kg (ip) 30 min prior to the test session (Plaza-Zabala et al., 2010). The selected dose of SB modulates the anxiogenic-like effects of nicotine (Plaza-Zabala et al., 2010) and regulates the reinstatement of nicotine-seeking behavior (Plaza-Zabala et al., 2013). Vehicle was delivered at the same volume as the SB solution.

### Behavioral Testing

#### Selection of HD/LD Subpopulations

At the beginning of the studies, animals of each cohort were first exposed to a single 2 h episode of DID, after which HD/LD subpopulations were identified and separated according to whether their EtOH intake was above or below the group median. For the DID procedure, 3 h into the dark cycle, each water bottle was replaced with a single bottle of 20% (v/v) EtOH, which was weighed and placed in the home cage for 2 h. During this time, food consumption and EtOH intake (g/kg/2 h) were recorded. Body weight (BW) was recorded every 4 days. An empty cage for each shelf was used for the placement of dummy bottles to measure lost fluid, which was subtracted from the total consumption as a control for fluid spillage.

#### Intermittent Drinking in the Dark (iDID)

The iDID procedure is a prolonged and intermittent version of the standard DID. On days 1, 3 and 5, all water bottles were replaced with an EtOH (20% v/v) solution for 2 h; on days 2, 4, 6 and 7, mice had food and water *ad libitum* but no access to EtOH. This 7-day cycle was repeated four times (see Figure 3). After that, a test day was administered using a 4 h session repeating the conditions described for the 2 h DID trials. EtOH intake was measured as g/kg/2 h.

**Figure 1:**
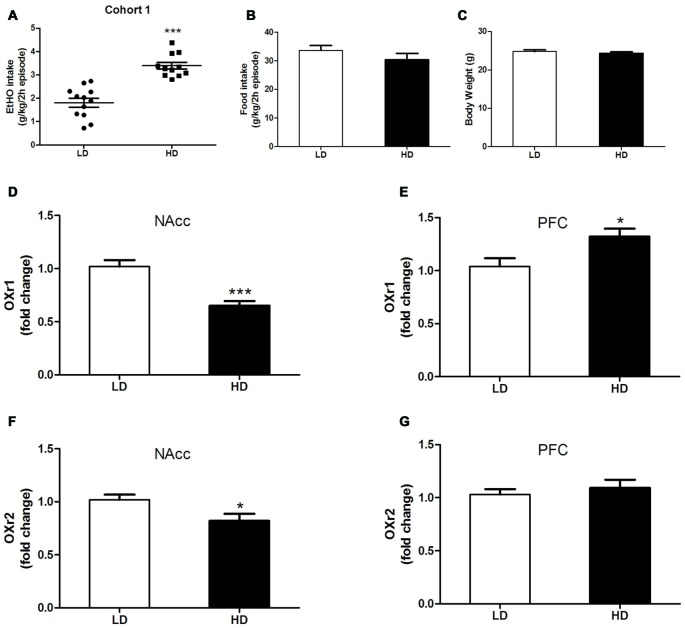
Individual consumption of 20% (v/v) ethanol (EtOH) **(A)**, food intake **(B)** and body weight (BW) **(C)** during a single 2 h episode of drinking in the dark (DID), from which high drinkers (HD) and low drinkers (LD) subpopulations were identified [Cohort 1]. OX1r **(D,E)** and OX2r **(F,G)** mRNA expression within the NAcc and the prefrontal cortex (PFC) in HD vs. LD subpopulations. Brains were extracted after 10 days without EtOH after the DID test. All values are means ± SEM. *p < 0.05 and ***p < 0.001.

**Figure 2:**
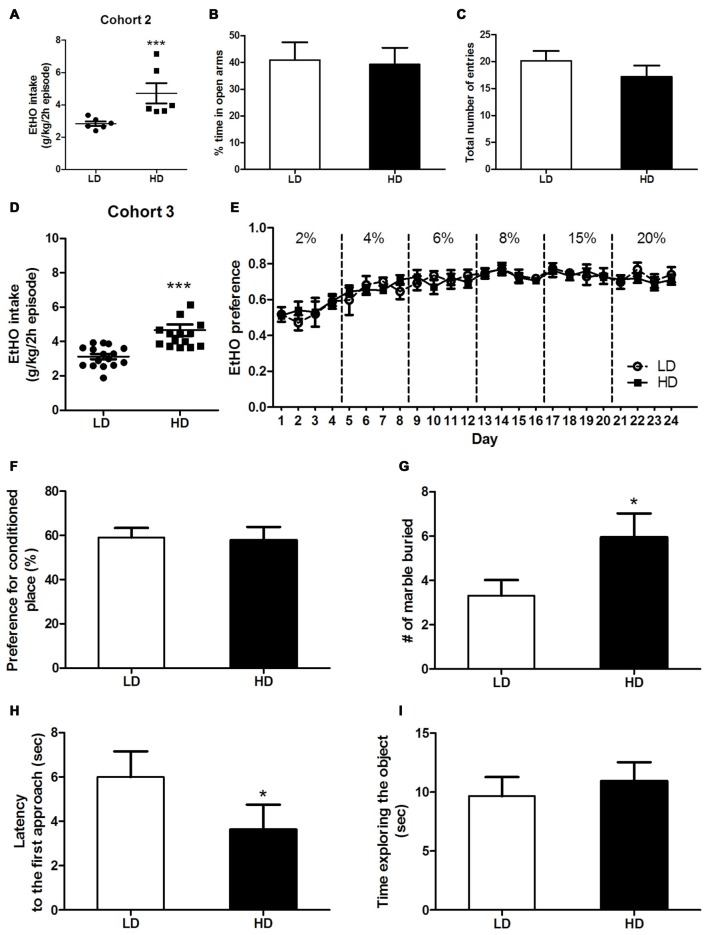
Individual consumption of 20% (v/v) EtOH during a single 2 h episode of DID, from which high vs. low drinkers (HD/LD) subpopulations were identified [Cohort 2] **(A)**. Basal anxiety-like responses in HD/LD, as measured by elevated plus maze (EPM) **(B,C)**. Individual consumption of 20% (v/v) EtOH during a single 2 h episode of DID, from which HD/LD subpopulations were identified [Cohort 3] **(D)**; EtOH preference data over 6 progressively increasing EtOH concentrations by HD/LD, in a 2-bottle free choice procedure **(E)**. Preference (%) for the chamber conditioned to EtOH after 4 EtOH conditioning trials in a conditioned place preference (CPP) procedure by HD/LD **(F)**. Number of marbles buried **(G)** latency to approach **(H)** and time exploring **(I)** a novel object in HD/LD subpopulations. Behavioral tests were conducted after 10 days without EtOH after the DID test. All values are means ± SEM. *p < 0.05 and ***p < 0.001.

**Figure 3:**
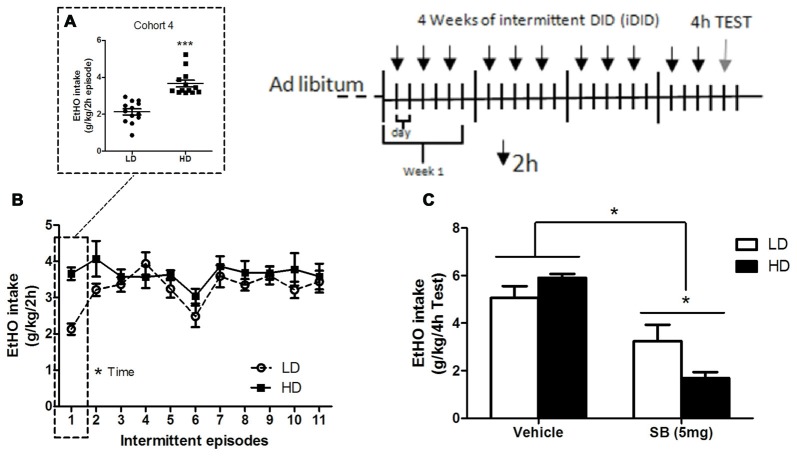
Individual consumption of 20% (v/v) EtOH obtained after a single 2 h DID episode, from which HD/LD subpopulations were identified [Cohort 4] **(A)**; consumption of 20% (v/v) EtOH by HD/LD mice during prolonged and intermittent exposure to DID episodes (iDID) **(B)**; consumption of 20% (v/v) EtOH by HD/LD subpopulations during a 4 h DID test following ip administration of SB (5 mg/kg) or vehicle **(C)**. All values are means ± SEM. *p < 0.05 and ***p < 0.001.

For the pharmacological challenge [Cohort 4], on day 12 following iDID, the animals were assigned to two groups matched for EtOH fluid consumption during the training period and then given an intraperitoneal (ip) injection of SB (5 mg/kg) or vehicle 30 min before EtOH presentation in the DID task. EtOH consumption was measured as g/kg/4 h.

#### Novel Object Exploration

This is a behavioral task that assesses reactivity to a novel object as an index of neophobia-related anxiety (Lee et al., 2015). Mice [Cohort 3] were placed in a white acrylic box (40 × 40 × 30) for three consecutive habituation days (30 min each day). Then, on day 4, a novel object was introduced in the cage, and the latency until the first approach to the object, the time the mouse spent investigating the object, and the number of contacts performed were all measured by a blind experimenter.

#### Marble Burying Test

This test has been used to measure anxiety-induced defensive burying and has also been employed as an index of compulsivity (Lee et al., 2015). Mice [Cohort 3] were placed in a white acrylic box (25 × 50 × 15) in which opaque marbles (20) had been placed on top of fresh bedding. The number of marbles with two-thirds of their surface covered by the mouse was scored as buried during a 30 min test session (Angoa-Pérez et al., 2012).

#### Elevated Plus Maze

The EPM is a widely used behavioral task that measures anxiety-like responses in rodents (Walf and Frye, 2007). The EPM apparatus used in this experiment was made up of two open and two enclosed arms (30 cm × 5 cm, and 30 cm × 15 cm × 5 cm, respectively) extending from a common central square (5 cm × 5 cm) and elevated 50 cm above floor level on five pedestals (Cibertec, S.A., Madrid, Spain). The maze floor and walls were made of black Plexiglas. The open arms lacked any wall or rims. Indirect halogen illumination provided 210 lux onto the open arms and 75 lux onto the closed arms. The EPM task was carried out as described elsewhere (e.g., Viosca et al., 2009). Briefly, the animals [Cohort 2] were acclimated to the experimental room for 1 h during two consecutive days. On day 3 (test day), mice were placed in the center of the maze facing an open arm, and their behavior was automatically recorded (Cibertec Software, S.A., Madrid, Spain) for 5 min using photo beams installed along the chamber walls. The percentage of time spent in open/closed arms and the total number of entries were recorded.

#### Voluntary EtOH Consumption in a Two-Bottle Free Choice Procedure

This study aimed to determine the relative preference of HD vs. LD mice for EtOH solutions of progressively increasing concentrations. One new cohort of mice [Cohort 3] first underwent one single episode of binge-like EtOH drinking (2 h) that segregated HD and LD subpopulations, followed by a period of 10 EtOH-free days during which they received food and water *ad libitum*. Then, mice underwent a two-bottle free choice procedure, with 24 h unlimited access to EtOH and water in their home cages as follows: one bottle always contained water, and the other contained a solution of EtOH at progressively increasing concentrations (2%, 4%, 6%, 8%, 15%, 20%). Each EtOH concentration was tested for 4 days. Water and EtOH solutions were exchanged daily, and fluid consumed (ml) was recorded every 24 h. Placement of water and EtOH bottles (left vs. right) was counterbalanced to avoid side preference. Food was available *ad libitum*, and mice were weighed every 4 days. The ratio of EtOH preference was determined (EtOH preference = EtOH intake/EtOH intake + water intake).

#### EtOH Conditioned Place Preference

An EtOH CPP task evaluated the reinforcing properties of EtOH [Cohort 3] (Ortega-Álvaro et al., 2015). The CPP apparatus consisted of two chambers of 30 × 30 × 30 cm (one with black walls and a coarse floor, and the other with black-dotted white walls and a smooth, plasticized floor) separated by a movable panel. Photo beam detectors installed along the chamber walls were used to register total time spent in each chamber (Cibertec Software S.A., Madrid, Spain). The CPP procedure consisted of three phases (Roger-Sánchez et al., 2012): (a) the first was the preconditioning phase (Pre-C), during which mice had free access to both compartments of the apparatus for 900 s on three consecutive days. On day 3, the time spent by the animal in each compartment during a 900 s period was recorded. During this phase, animals showing strong unconditioned aversion or preference for either compartment were discarded from the study (33% for white/67% for black compartment); (b) next was the conditioning phase: animals were conditioned to one compartment with EtOH (2.5 g/kg at a volume of 0.2 ml/g, ip) through four consecutive EtOH-context pairings. Rodents received EtOH or vehicle immediately prior to being confined in the drug- or saline-paired compartment for 10 min. The central area was never accessible during conditioning, as it was blocked by guillotine doors; and (c) during the third phase, post-conditioning (Post-C), which took place on day 12, the guillotine doors were removed, and during a test step of 900 s, time spent by drug-free mice in each compartment was recorded.

### Orexin Receptor 1 (OX1r) and 2 (OX2r) Gene Expression Analyses by Real-Time PCR

Ten days after undergoing a single episode of binge-like EtOH drinking, OX1r/OX2r mRNA expression within the PFC and the NAcc was analyzed. Total RNA was isolated from frozen (−80°C) NAcc and PFC micro punches with Tri Reagent (Ambion) and subsequently retrotranscribed to cDNA. Gene expression of OX1R and OX2r in the NAcc and PFC was assessed using real-time PCR in HD or LD mice. Quantitative analysis of gene expression was measured with the following TaqMan^®^ Gene Expression assays: “Mm01185776_m1” for OX1R and “Mm01179312_m1” for OX2r (Life Technologies, Madrid, Spain). Real-time PCR experiments were performed on the StepOne Plus system (Life Technologies, Madrid, Spain), and the reference gene used was Rn18S rRNA, detected using TaqMan^®^ ribosomal RNA control reagent “Mm03928990_g1”. Data for each target gene were normalized to the endogenous reference gene, and the fold change in target gene mRNA abundance was determined using the 2^−∆∆Ct^ method (Livak and Schmittgen, 2001).

### Data Analysis

All data in this report are presented as the means ± SEM. In all the studies presented, HD/LD subpopulations were segregated based on the median of EtOH consumption scores obtained during the first session of a DID procedure. Independent analyses of variances (ANOVAs) and Student’s *t*-tests were then used to analyze all data recorded in the study. Significance for all tests was set at *p < 0.05 and ***p < 0.001. When significant interactions emerged, *post hoc* analyses were performed using Newman–Keuls (NK) tests. Grubb’s test was used to identify and exclude outlier data.

## Results

### OX1R and OX2R Expression within the Prefrontal Cortex (PFC) and the Nucleus Accumbens (NAcc) in HD vs. LD Subpopulations

Figures 1A–C shows data representing 20% (v/v) binge-like EtOH drinking (A), food consumption (B) and BW (C) observed in HD vs. LD subpopulations that spontaneously emerged during a single 2 h DID session [cohort 1]. Independent one-way ANOVAs performed on EtOH DID consumption, food intake and BW measures obtained in HD and LD mice revealed that LD mice consumed less EtOH than did HD mice (*F*_(1,21)_ = 42.10; *p* < 0.001; Figure 1A). However, in the first episode of a DID test, no subpopulation differences were found in food consumption (*F*_(1,21)_ = 1.28; *p* = 0.27; Figure 1B) or in BW measures (*F*_(1,21)_ = 0.63; *p* = 0.46; Figure 1C).

Correlated measures of OX1r/OX2r mRNA expression in the PFC and NAcc in HD vs. LD subpopulations are shown in Figures 1D–G. We report significantly lower OX1r mRNA expression in the NAcc of HD mice (Figure 1D, Student’s *t*-test, *t* = 4.113; *p* < 0.001, *df* = 22) than in the LD subpopulation. However, HD mice showed significantly higher OX1r mRNA expression in the PFC than did the LD subpopulation (Figure 1E, Student’s *t*-test, *t* = −2.687; *p* < 0.05, *df* = 22). Regarding regional OX2r mRNA expression, compared to LD animals, HD mice showed significantly lower OXR2 mRNA expression in the NAcc (Figure 1F, Student’s *t*-test, *t* = 2.434; *p* = 0.02, *df* = 22). Finally, no subpopulation (HD vs. LD) differences were observed in OXr2 mRNA expression in the PFC (Figure 1F, Student’s *t*-test, *t* = −0.672; *p* = 0.51, *df* = 22).

### Evaluation of Basal Anxiety-Like Responses, EtOH Preferences and Sensitivity to the Reinforcing Properties of EtOH in HD vs. LD Subpopulations

High basal anxiety and increased sensitivity to the reinforcing properties of EtOH have all been widely proposed to play key roles in vulnerability to EtOH addiction (Wand, 2005; Iacono et al., 2008; Casey and Jones, 2010).

As showing in Figures 2A,D, HD/LD subpopulations in cohorts 2 and 3 were segregated by a single 2 h EtOH binge-DID episode according to whether their EtOH intake was above or below the group median. Independent one-way ANOVAs performed on EtOH consumption of cohort 2 (*F*_(1,10)_ = 8.52; *p* < 0.001; Figure 2A) and cohort 3 (*F*_(1,29)_ = 18.64; *p* < 0.001; Figure 2D) again showed that LD mice consumed less EtOH than HD mice.

Ten days after undergoing a single episode of binge-like EtOH drinking (2 h), these cohorts were evaluated for the following: (a) different types of basal anxiety-like responses as measured by a plus-maze test (cohort 2, Figures 2B,C), the marble-burying and novel object exploration tests (Cohort 3, Figures 2G–I); (b) sensitivity to the reinforcing properties of EtOH as measured by the 2-bottle choice task and a conditioned EtOH place-preference test (cohort 3, Figures 2E,F, respectively).

#### Anxiety-Like Response in HD vs. LD Mice as Measured by Elevated Plus Maze

Independent one-way ANOVAs performed on the percentage of time in open arms (*F*_(1,10)_ = 0.34; *p* = 0.86; Figure 2B) and the total number of entries (*F*_(1,10)_ = 1.18; *p* = 0.31; Figure 2C) revealed no statistically significant subpopulation differences in basal anxiety or locomotor activity, respectively, in HD vs. LD mice.

#### EtOH Preferences and Sensitivity to the Reinforcing Properties of EtOH in HD vs. LD Mice as Measured Respectively by a 2-Bottle Choice Task and a Conditioned EtOH Place Preference Test

A repeated-measures ANOVA (2 × 24), (subpopulation × time) performed on voluntary EtOH consumption observed in a 2-bottle choice task during 24 days (Figure 2E) revealed no statistical significance for subpopulation and time as main factors, and there were also no significant interactions of factors (HD/LD EtOH preference: (*F*_(1,10)_ = 0.09; *p* = 0.93); time (*F*_(23,230)_ = 7.65; *p* < 0.01); subpopulation × time interaction (*F*_(23,230)_ = 0.6; *p* = 0.98; Figure 2C). Similarly, a one-way ANOVA performed on EtOH place-preference measure (Figure 2F) revealed no subpopulation differences (*F*_(1,12)_ = 0.02; *p* = 0.88). Taken together, these data suggest that HD and LD mice show similar sensitivity to the reinforcing properties of EtOH.

#### Anxiety-Like Responses in HD vs. LD Mice as Measured by the Marble-Burying Test and the Novel Object Exploration Task

A one-way ANOVA performed on measures obtained in the marble-burying test (Figure 2G) showed that HD mice buried significantly more marbles than LD mice (*F*_(1,30)_ = 4.38; *p* < 0.05), indicating higher levels of anxiety behavior in the HD subpopulation (Figure 2G). Additionally, the ANOVA conducted on the latency to first approach (*F*_(1,30)_ = 5.73; *p* < 0.05; Figure 2H) and time spent exploring (*F*_(1,30)_ = 0.31; *p* = 0.59; Figure 2I) measures obtained during the novel object exploration test revealed that HD mice showed shorter latencies than LD mice to the first approach to a novel object.

### Effects of the Selective OX1r Antagonist SB-334867 (5 mg/kg) in HD vs. LD Subpopulations Following 12 Intermittent Episodes of EtOH Binge Drinking (iDID)

Mice from cohort 4 were analyzed for binge-like drinking of 20% (v/v) EtOH in HD vs. LD subpopulations that emerged during one single 2 h DID episode (Figure 3A); intermittent binge-like consumption of 20% (v/v) EtOH during the iDID procedure (Figure 3B) and EtOH consumption during the 4 h-test day in response to intraperitoneal (ip) injection of SB (5 mg/kg; Figure 3C).

Independent one-way ANOVAs performed on EtOH consumption data for HD/LD populations in cohort 4 (*F*_(1,24)_ = 39.22; *p* < 0.001; Figure 3A) demonstrated that LD mice consumed significantly less EtOH than did HD mice. An additional repeated measures (2 × 10, subpopulation × time) ANOVA was conducted on binge-like EtOH consumption measures during the 10 successive 2 h-DID episodes (Figure 3B). Statistically significant differences were observed for time (*F*_(9,198)_ = 2.36; *p* < 0.05) but not for either subpopulation (*F*_(1,22)_ = 2.84; *p* = 0.11) main effects or for the subpopulation × time interaction (*F*_(9,198)_ = 0.74; *p* = 0.67), indicating that the HD and LD subpopulations achieved similar levels of EtOH binge-consumption over time.

An independent (2 × 2, subpopulation × treatment) ANOVA was conducted on binge-like EtOH consumption measures in response to administration of SB or vehicle on the 4 h-test day (day 12; Figure 3C). Statistical analyses showed that peripheral administration of 5 mg/kg of the selective OX1R antagonist SB significantly reduced EtOH intake (treatment: *F*_(1,23)_ = 40.61; *p* = 0.00) in both HD and LD animals. The subpopulation main factor did not reach statistical significance (*F*_(1,23)_ = 0.54; *p* = 0.47). Interestingly, the ANOVA revealed statistical significance for the subpopulation × treatment interaction (*F*_(1,23)_ = 6.33; *p* = 0.02). Additional NK *post hoc* analysis showed higher reduction in EtOH consumption in the HD mice in response to SB, compared to LD, which suggests a higher response to SB in HD mice.

## Discussion

The most important finding in this study was that a single episode of binge-like EtOH drinking in a 2 h DID session consistently differentiates two neurobehavioral endophenotypes, HD vs. LD, in inbred C57BL/6J mice, which predicts the response to a pharmacological challenge with a selective OX1r antagonist following intermittent EtOH binge-drinking. Molecular characterization of DID endophenotypes also revealed decreased basal OX1r and OX2r mRNA expression within the NAcc and increased OX1r within the PFC, in the HD endophenotype. No endophenotypes differences were found for EtOH preferences as tested at different concentrations or in sensitivity to the reinforcing properties of EtOH, as evaluated by the EtOH place conditioning task. Given that C57BL/6J mice are genetically identical, it is likely that HD/LD neurobehavioral endophenotypes might reflect an epigenetic mechanism, which is consistent with previous observations showing high phenotypic variation under identical environmental conditions in binge consumption of EtOH (Barkley-Levenson and Crabbe, 2014) and diet-induced obesity in male C57BL/6J inbred mice (Koza et al., 2006).

Our first remarkable observation was the existence of two subpopulations for DID, HD vs. LD, which consistently and spontaneously emerged during a single 2 h EtOH DID session, in all the studies conducted. The fact that HD/LD groups showed no correlative differences in BW or basal food consumption supports the hypothesis that HD/LD behavioral endophenotypes are unrelated to caloric consumption or energy balance. Moreover, we rule out endophenotype differences in sensorial gustative processing or EtOH metabolism based on data obtained in the EtOH preference study showing similar level of EtOH consumption in both populations, at different EtOH concentrations.

When further characterizing HD/LD endophenotypes for differences in a set of measures of anxiety-like behaviors, present results seems a little puzzling. Thus, compared to LD mice, we report in HD mice lower latency to approach to novel object indicative of reduced neophobia; higher marble burying, which might support higher anxiety-induced defensive burying behavior and no differences in basal anxiety as measured by EPM. Given that total arm entries in the EPM and total time exploring the novel object showed no endophenotype differences, we rule our group differences in basal locomotor behavior. First, data collected in the reaction to novelty test are consistent with basal endophenotype differences in neophobia to the novel EtOH when first presented. In this regard, we cannot conclude whether HD/LD endophenotypes are primary and specific for EtOH intake or rather they reflect a secondary trend for high/low consumption of new substances primarily driven by endophenotype differences for novelty seeking. Second, because the marble burying test has been considered to be sensitive to impulsivity/compulsivity traits (Llaneza and Frye, 2009; Angoa-Pérez et al., 2012), there is the possibility that increased marble burying in HD mice indicates higher basal compulsivity trait rather than anxiety-induced defensive burying. Consistent with this idea, novelty-seeking and compulsivity/impulsivity traits are both risk factors associated with behavioral addictions (Black et al., 2015) and are predictive endophenotypes associated with compulsive consumption (Belin-Rauscent et al., 2016). Because DID represents a procedure for successfully modeling binge drinking in humans during the early stages in the EtOH addiction cycle (Cox et al., 2013; Thiele and Navarro, 2014), our observation that this procedure consistently segregates two EtOH DID endophenotypes showing specific traits differences might contribute to understanding key factors such as increased compulsivity, reduced neophobia and/or different coping strategies emerging in the early stages of the addiction cycle, favoring vulnerability to excessive binge-like EtOH consumption.

Our present study also addressed a molecular characterization of HD/LD endophenotypes focused on the OX system. The second important observation is that, compared to LD mice, HD mice showed decreased basal OX1r and OX2r mRNA expression within the NAcc and increased OX1r mRNA expression within the PFC. It is unlikely that changes in brain mRNA expression elicited by differences in total EtOH volume consumed and/or EtOH concentration reaching the brain during the 2 h DID initial episode might persist long term, 10 days later, when brains were extracted for evaluation. Moreover, we have previously reported similar regional mRNA expression as a result of a single 2 h episode of sucrose, EtOH and saccharin DID, in which the amount of sucrose consumed was almost double that of saccharin or EtOH. Thus, we conclude that total volume ingested in a DID test was unrelated to the expression of mRNA OX receptors in the brain (Alcaraz-Iborra et al., 2014; Carvajal et al., 2015). Because intracellular metabolic factors such as post-transcriptional phenomena could account for differences between gene transcription and final protein expression (Maier et al., 2009) suggesting that gene and protein expression changes could be even opposite, we cannot definitively conclude whether present data are indicative of increased OX1 receptors and OX activity within the PFC and reduced OX1r/OX2r and OX activity within the NAcc. Nonetheless, there is the exciting idea that basal differences in regional expression of mRNA OX receptors in the HD and LD endophenotypes might represent an innate neurobiological molecular feature indicating reduced/elevated numbers of OX receptors and possibly OX activity in the NAcc and PFC, respectively. Whether the observed molecular differences in OX mRNA are causally linked to physiological and/or behavioral traits favoring increased vulnerability to EtOH binge drinking remains unclear, but some ideas are discussed below.

First, endophenotype differences in OX1r/OX2r mRNA expression within the NAcc, a limbic region involved in EtOH drinking, EtOH-induced CPP and EtOH-induced psychomotor sensitization in mice (Bahi and Dreyer, 2012), were not accompanied by differences in HD/LD EtOH preference or EtOH CPPs, which might suggest that OX activity within the NAcc has no a key role in modulating sensitivity to EtOH reinforcing and toxic properties. Moreover, our present data are in accordance with previous pharmacological studies showing that systemic blockade of OX1 receptors plays no role in EtOH-induced place preference behavior (Voorhees and Cunningham, 2011). On the other hand, we cannot rule out a role for OX in EtOH seeking since a growing experimental pharmacological and genetic literature has supported over the last years a role for OX in EtOH seeking (Lawrence et al., 2006; Brown and Lawrence, 2013; Martin-Fardon and Weiss, 2014) and EtOH self-administration (Dhaher et al., 2010; Jupp et al., 2011; Brown et al., 2013). Thus, in a 2-bottle free choice paradigm, peripheral administration of the selective OX1r antagonist SB-334867 significantly reduced voluntary EtOH consumption in Sprague-Dawley rats showing high, but not low, EtOH preference (Moorman and Aston-Jones, 2009). Further, OX1r antagonism/blockade reduced voluntary EtOH self-administration in rats selectively bred for high EtOH preference (Anderson et al., 2014) and EtOH binge-drinking in C57BL/6J mice (Anderson et al., 2014; Carvajal et al., 2015; Olney et al., 2015). Taking into account first, the particular function of OX1r within the medial NAcc Shell, a key region involved in EtOH consumption in both mice and rats (Lei et al., 2016), and second, previous literature showing a role for OX activity in high EtOH drinkers, additional studies combining regional OX molecular characterization with appetitive, rather than consummatory behavior testing, will help to understand how endophenotype differences in NAcc mRNA OXr expression links to potential endophenotype differences in EtOH seeking.

Second, the proposed idea that increased number of buried marbles in HD mice support higher levels of compulsive/impulsive digging behavior (Angoa-Pérez et al., 2012) in the HD endophenotype is consistent with previous clinical and experimental data linking OX activity to compulsivity/impulsivity traits and binge EtOH consumption. Thus, clinical (Fillmore, 2003; Tarter et al., 2003; Sanchez-Roige et al., 2014) and experimental evidence (Dawe and Loxxton, 2004; Velázquez-Sánchez et al., 2014) indicate that high trait impulsivity predisposes to binge-like consumption of rewarding stimuli such as palatable food, drugs and EtOH. Interestingly, OX signaling modulates compulsive-driven consumption of palatable food, EtOH and drugs (Boutrel et al., 2010; Merlo Pich and Melotto, 2014). On the other hand, anatomical studies in mice (Horst and Laubach, 2013; Parent et al., 2015), rabbits (Hattori et al., 2014), primates and human (Dixon et al., 2014), have reported functional subdivisions in the mPFC, where Prelimbing cortex (PrL) is mainly associated with emotion-related cognitive processes and behavioral flexibility and the Infralimbic cortex (IL) is essential for controlling impulsivity (Chudasama et al., 2003). Interestingly, even though little is known about the OX innervation pattern, the density of the orexinergic fibers increases from the rostral part to the caudal part of the mPFC, regardless of AC, PL or IL, suggesting a rostro-caudal hierarchy of LH orexinergic innervation to the mPFC (Jin et al., 2016). Taking together anatomical evidence of OX innervation of mPFC subregions (Jin et al., 2016), the role of the mPFC as a key brain region involved in compulsivity/impulsivity (Fuster, 2001; Ambar and Chiavegatto, 2009), and pharmacological evidence linking OX to binge-drinking, we speculate that elevated basal OX activity in the IL area of the mPFC of the HD endophenotype might be linked to increased compulsivity/impulsivity and, consequently, spontaneous elevated EtOH binge-drinking in HD mice (Alcaraz-Iborra and Cubero, 2015).

Apparently contradictory with the idea linking high EtOH intake in HD to high impulsivity and OX activity, we report here that LD mice spontaneously increased EtOH binge drinking matching the same level of EtOH consumption by HD, after three episodes of iDID. The observation that intermittent exposure to EtOH binge-drinking triggered a rapid increase in EtOH consumption in LD mice might suggest that intermittent EtOH DID exposure might work as a key factor favoring OX activity in LD mice, leading to elevated EtOH binge-consumption over time. In agreement with it, intermittency has been shown to trigger high EtOH consumption in rat strains not prone to drink high levels of EtOH (Simms et al., 2008); moreover, we have recently found that continued but not intermittent DID exposure keeps endophenotypes differences in EtOH consumption stable over time (unpublished data from our lab). Noteworthy, even though LD matched HD EtOH intake over time, a pharmacological test uncovered the existence of silent endophenotype differences in the OX system. Thus, when challenged with 5 mg/kg of the selective OX1r antagonist SB, LD mice were significantly less sensitive to the inhibitory effect of the compound on EtOH binge drinking, indicating higher OX1r functionality in this group. Taking together present data in LD mice, either basal increased OXr1 in the NAcc of LD mice explain their reduced sensitivity to OXr1 blockade in the pharmacological challenge, and/or repetitive EtOH iDID triggered increased OXr1 functionality in other brain region/s over time in LD endophenotype. Consistent with the idea, it has been proposed that binge-like EtOH drinking in DID procedures may be modulated by transient changes in central neurochemical systems that arise from high BECs in vulnerable, non-dependent animals (Thiele and Navarro, 2014). Such initially transient neuroplastic changes might become consolidated during repeated episodes of binge-like drinking, contributing to the transition to addictive states (Cox et al., 2013; Thiele and Navarro, 2014). According to that idea, a possibility is that because C57BL/6J mice are a strain showing vulnerability to develop high EtOH consumption, intermittent exposure might have triggered increased EtOH DID in LD which in turn, increased OX activity in the NAcc and/or PFC in a positive loop. On the other hand, a ceiling effect preventing any additional increase and escalation of EtOH DID over time might explain molecular, behavioral and pharmacological differences in HD mice related to LD. In any case, whether basal, EtOH-triggered endophenotype differences or both combined, we underscore present pharmacological results confirming the ability of the original HD/LD separation to predict EtOH consumption responses to OXr1 blockade and reinforcing the interest in considering the existence of HD/LD endophenotypes in DID studies.

The present data reinforce three main ideas: first, the DID model is valid for exploring neurobehavioral processes underlying the early stages of the addiction cycle. Second, the DID task consistently segregates two behavioral endophenotypes; further evaluation will determine whether those endophenotypes specifically correlate with increased OX1r functionality within the PFC or other unexplored brain regions. Third, the HD and LD neurobehavioral endophenotypes can predict pharmacological responsivity to the selective OX1r antagonist SB. In light of the available data showing the importance of endophenotypes and their interactions with drug addiction (Walley et al., 2009; Kalueff et al., 2015), analysis of the HD and LD endophenotypes segregated by the DID procedure might help to understand the neurobehavioral mechanisms underlying early stages of EtOH binge-drinking disorders.

## Author Contributions

IC provided the overall coordination and supervision for the whole study. IC and MA-I were responsible for the study concept and design. MA-I conducted behavioral characterization. MA-I and ER-O carried out pharmacological studies. FN and JM were responsible for real-time PCR analysis. LF was responsible for statistical analyses. IC and MA-I wrote the manuscript. All authors critically reviewed the content and approved the final version for publication.

## Conflict of Interest Statement

The authors declare that the research was conducted in the absence of any commercial or financial relationships that could be construed as a potential conflict of interest.
